# Modeling T Cell Fate

**DOI:** 10.1146/annurev-immunol-101721-040924

**Published:** 2023-04-26

**Authors:** Rob J. De Boer, Andrew J. Yates

**Affiliations:** 1 Theoretical Biology and Bioinformatics, Department of Biology, Utrecht University, Utrecht, The Netherlands;; 2 Department of Pathology and Cell Biology, Columbia University Irving Medical Center, New York, NY, USA;

**Keywords:** T cells, fate determination, mathematical modeling

## Abstract

Many of the pathways that underlie the diversification of naive T cells into effector and memory subsets, and the maintenance of these populations, remain controversial. In recent years a variety of experimental tools have been developed that allow us to follow the fates of cells and their descendants. In this review we describe how mathematical models provide a natural language for describing the growth, loss, and differentiation of cell populations. By encoding mechanistic descriptions of cell behavior, models can help us interpret these new datasets and reveal the rules underpinning T cell fate decisions, both at steady state and during immune responses.

## 1. INTRODUCTION

The expansion and differentiation of antigen-specific T cells from naive or memory precursors following infectious challenge, and the establishment or supplementation of diverse and persistent memory populations, are defining features of adaptive immunity in vertebrates. These memory populations can be classified most broadly as either circulating and tissue-resident, but each in turn comprises subpopulations with different effector capabilities and capacities for self-renewal or differentiation. Reaching a consistent and functionally meaningful classification of these subsets is an ongoing challenge (1–4). Diversification of T cell fates is linked to cell division (5–8), suggesting that memory emerges from small precursor populations in branched structures, shaped and pruned by differentiation, proliferation, senescence, and cell death. However, for many years our understanding of the pathways and dynamics of commitment to different memory lineages, and how they are subsequently maintained, was hampered by the constraint of observing T cell populations cross-sectionally, and in bulk.

The ability to track the development of the T cell response from a single precursor triggered a wave of studies that explored how phenotypic heterogeneity emerges (9, 10). Many of these studies involved mathematical modeling, for perhaps three reasons. The first is that mathematical models are particularly useful for describing the complex and sometimes nonintuitive population dynamics that can emerge from the interplay of the basic processes of cell growth, death, and differentiation, and they provide a way to encode the rules governing these processes in a mechanistic way. The second is that statistical tools exist for quantifying our degrees of belief in different models of cell behavior, and thus for weighing the evidence for alternative biological mechanisms. These tools typically involve a trade-off between the descriptive power of a model and its complexity or flexibility. Third, mathematical models fitted to data typically yield estimates of key quantities such as interdivision times, cell life spans, and probabilities of commitment to different lineages. These estimates, usually referred to as parameters, reinforce our mechanistic understanding of immune responses. They can also serve as a sanity check—a model that fits data well but yields biologically implausible parameters can be rejected. Thus, mathematical models can both help formalize our intuition and act as handrails to guide us along the path of testing and rejecting hypotheses.

In this review we focus on how mathematical modeling has helped us interpret the data from fate-mapping experiments. We begin with the dynamics of naive and memory T cells at steady state and then address the emergence of phenotypic diversity in T cell responses.

## 2. MODELING THE FATES OF NAIVE AND MEMORY T CELLS IN HEALTH

Much of what we know about life spans and rates of self-renewal of T cell subsets under healthy, steady-state conditions derives from DNA-labeling assays, in which dividing cells acquire a traceable label, such as bromodeoxyuridine (BrdU) or deuterium. The time courses of the numbers or proportions of labeled cells within a population during and after the administration of the label contain information regarding rates of division and turnover, and potentially any heterogeneity in these dynamics within the population (11). (Henceforth we define turnover to mean loss of cells from a population through death or differentiation. If a population’s size is constant, its total rate of turnover, as cells lost from the population per day, equals the rate of production of new cells by influx and division.) Mathematical models are required to fully interpret these data. More challenging perhaps is to extend our descriptions of these population dynamics to capture the process of differentiation. To do this, one needs to identify cohorts of precursors and observe the population dynamics and fates of their descendants, either by longitudinal sampling or by cross-sectional analyses of cohorts at later times. Here we discuss several experimental approaches to studying T cell life histories, and how mathematical models have helped us to increase the information we can glean from them.

### 2.1. Quantifying T Cell Development Using Chimeric Mouse Models

**Figure 1:**
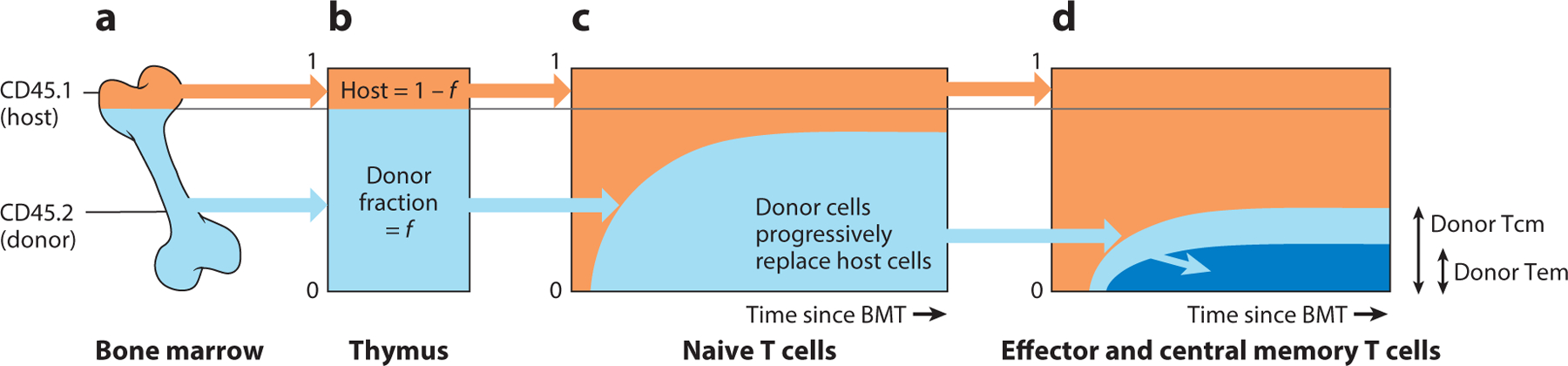
Using chimeric mice to explore the topology and kinetics of T cell differentiation of peripheral T cell subset at steady state. (*a*) Stable chimerism in bone marrow is achieved rapidly after busulfan treatment and HSC transplantation, leading to a fraction *f* of HSCs being donor derived. (*b*) Stable chimerism at all stages of thymic development is reached within four to six weeks, at the same level, *f*, as bone marrow. (*c*) The time course of accumulation of naive donor T cells is determined by the turnover rate(s) of naive T cells, and of the donor chimerism in the thymus. The observed shortfall in replacement implies that influx from the thymus wanes strongly with time and/or that older (host) cells persist longer on average than newer (donor) cells (13). (*d*) In this example, donor Tcm (*light blue*) accumulate faster than donor Tem (*dark blue*), suggesting that Tcm turn over more rapidly. The higher level of stable chimerism among Tcm suggests that any waning of influx, or differences in persistence with cell age, are more pronounced for Tem. In addition, the consistently higher level of chimerism among Tcm is compatible with a Tcm→Tem differentiation pathway (*blue arrow*) but excludes the reverse possibility. The replacement kinetics illustrated here have been observed among memory CD4 T cells in mice (15). Abbreviations: BMT, bone marrow transplantation; HSC, hematopoietic stem cell; Tcm, central memory T cell; Tem, effector memory T cell.

Bone marrow chimeric mice, in which host and donor cells can be distinguished by congenic markers, provide a means of following the developmental trajectories of new, donor-derived cells within the recipient (host). Typical transplant-conditioning regimes, such as irradiation or antibody-mediated cell depletion, create significant physiological perturbations in the host that disrupt normal homeostasis. One system that attempts to sidestep this issue, and allows us to observe cell fates in mice under healthy physiological conditions, uses low doses of the transplant-conditioning drug busulfan to specifically deplete hematopoietic stem cells (HSCs) (12). HSCs are then reconstituted with congenic, T cell–depleted bone marrow (Figure 1a). During this process the mice remain healthy and peripheral lymphocyte subsets remain intact. The transplant rapidly replenishes T and B cell progenitor populations, and stable chimerism at all stages of thymic development is achieved by six weeks after transplantation (13) (Figure 1b). One can then observe donor-derived lymphocytes populate peripheral subsets, which were previously replete with host-derived cells (Figure 1c,d). The sequence and kinetics with which this infiltration occurs tell us about the lineage relationships between T cell subsets, and self-renewal and turnover within them. For example, in a unidirectional chain or tree of differentiation steps, in which at each step host and donor cells follow identical rules of behavior, one would expect that, given sufficient time, the chimerism in all lineages would equilibrate at the level of their common progenitor population. Any departure from these dynamics is informative. For example, a failure of donor-derived cells to populate a given cell subset to the expected level could derive simply from a decline in the total rate of flow of host and donor cell into it, with age. One can show that if the rate replenishment of a particular population from a precursor declines faster than the population average rate of turnover, it may never be replaced to the extent of the precursor (13). A shortfall in replacement could also imply that, on average, older (host-derived) cells have a fitness advantage over immigrant (donor-derived) cells. Further, the shape of the replacement curve is informative; a multiphasic approach to steady-state chimerism is suggestive of so-called kinetic heterogeneity (14), in which a cell subset comprises two or more subpopulations with different rates of division and turnover.

#### 2.1.1. Modeling naive T cell fates.

In a series of studies, busulfan chimeras were used to explore the dynamics of naive CD4 and CD8 T cells (13, 16, 17). Mathematical models of influx from the thymus, division, and loss were fitted to the time courses of replacement of host-derived naive T cells with donor cells. The investigators concluded that both naive CD4 and CD8 T cells in mice have an expected life span of a few weeks immediately following release from the thymus, divide very rarely, and exhibit a slow but progressive increase in their survival capacity with their post-thymic age. This behavior gives host-derived naive T cells, which are older on average than donor counterparts, a competitive advantage, and so explains the failure of naive donor T cells to replace host cells to the expected level (13) (Figure 1c). Hogan et al. (13) showed that this shortfall could not be explained solely by the waning production of new naive T cells (recent thymic emigrants) due to thymic involution. The ability of older naive T cells to persist longer than younger ones is consistent with other experimental studies (18, 19) and can also explain the slowing kinetic of loss of naive T cells following thymectomy, which has also been ascribed to a density-dependent regulation of cell numbers (20).

#### 2.1.2. Constitutive production of memory T cells.

Studies of busulfan chimeras also revealed that donor T cells accumulate slowly within circulating central memory (Tcm, defined as CD44^hi^CD62L^hi^) and effector memory (Tem, CD44^hi^CD62L^lo^) populations in the absence of overt infection (15, 21) (Figure 1d). Thus, memory-phenotype (MP) T cells are generated constitutively throughout the mouse life span. It is unclear whether these cells are truly antigen experienced or are naive T cells masquerading as memory cells, but there is evidence that they support pathogen-specific responses (22). Following the transplantation of congenically labeled thymi into healthy mice, donor-derived MP cells appear in the periphery almost in tandem with donor-derived naive T cells (23), indicating that at least some MP cells derive directly from recent thymic emigrants. This observation might be explained either by a subset of naive T cells being preprogrammed to acquire a memory phenotype, e.g., by being stimulated by self-antigens, or by newly exported naive T cells specific for environmental or commensal antigens being rapidly stimulated. Evidence for foreign antigen as a driver comes from another study modeling data from busulfan chimeras and quantifying the rate of memory production in specific-pathogen-free mice housed under different conditions (15). Mice in open cages with nonirradiated drinking water exhibited a greater flux into memory early in life, although not in adulthood, and maintained higher numbers of CD4 Tcm and Tem than those in individually ventilated cages with irradiated water. Germ-free mice also generate MP cells (15, 24), although at even lower levels, suggesting a role for self-antigens, although these mice are not entirely free of foreign antigens. Together, these results suggest that the antigenic burden in the environment contributes to establishing MP cells in neonates but that self-antigens and/or ubiquitous environmental antigens drive their production, and possibly maintenance, later in life.

### 2.2. Time-Stamping as a Means of Tracking Fate

Inducible reporter constructs allow the heritable stamping of cohorts of cells and following their trajectories in vivo. For example, a mouse model using a tamoxifen-inducible Cre recombinase driven by the CD4 promoter (25) has been used to study the fates of naive CD8 T cells after release from the thymus. In this model, a pulse of tamoxifen permanently induces red fluorescent protein (RFP) in cells expressing CD4, including CD4^+^CD8^+^ double-positive thymocytes. The cohort of naive CD8 T cells deriving from these labeled CD4^+^CD8^+^ precursors, and their descendants, continue to express RFP in the periphery. Reynaldi et al. (26) performed serial sampling of blood from these CD4 reporter mice pulsed briefly with tamoxifen at different ages. These experiments generated longitudinal estimates of the size of the surviving cohorts of naive CD8 T cells, allowing them to study the effects of both mouse and cell age on their dynamics. By comparing the statistical support for a set of descriptive mathematical models, they inferred that the net loss rate of naive CD8 T cells declines with the time since their release from the thymus. These kinetics might derive from a selective effect in which naive CD8 T cells are intrinsically heterogeneous with respect to their survival capacity, and longer-lived cells are selected for over time. Alternatively the increase in average survival might reflect an adaptation process, in which the propensity for survival rises cell-intrinsically with post-thymic age, as described above. This dataset was also notable in that individual mice were sampled repeatedly. Such data allow one to accommodate variations across mice in parameters, boosting our ability to discriminate between different models of the underlying biology. A reanalysis of these data with a more mechanistic model reiterated that naive CD8 T cells gradually increase their rates of loss through death or differentiation with their post-thymic age and are lost at higher baseline rates in neonates (17). These time-stamp mice were also used to demonstrate that fetal- and adult-derived naive T cells respond differently to antigenic challenge, both in the kinetics of their responses and in the effector and memory phenotypes they generate (27).

### 2.3. Modeling the Dynamics of Memory Maintenance

Persistence of antigen-specific T cell memory requires either that individual T cells be long-lived or that T cell clones persist dynamically, balancing loss with self-renewal (28). Such dynamic maintenance might also be supported with influx from a less-differentiated and possibly self-renewing, stem-like memory precursor (29, 30). To understand how the clonal structure and phenotype of antigen-specific memory evolves following the resolution of an immune response, we need to build and parameterize models of the differentiation, self-renewal, and attrition of these cell populations.

#### 2.3.1. Heterogeneity in turnover within memory.

**Figure 2:**
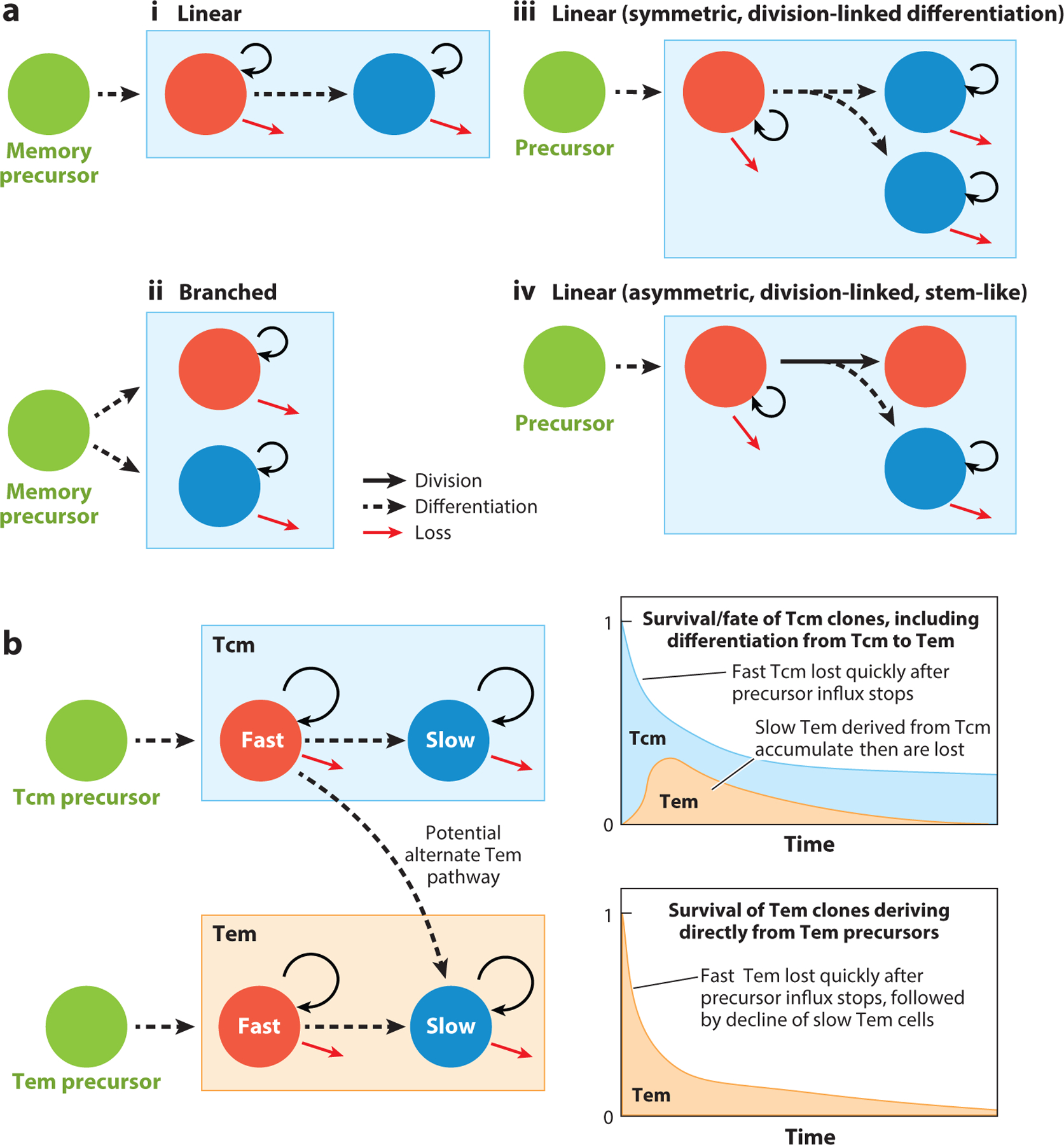
Modeling modes of memory T cell renewal, and of the survival and differentiation of memory clones. (*a*) Four potential modes of memory maintenance, illustrated with two phenotypically distinct subsets, are shown in red and blue. (*i*) Sequential differentiation, not linked to cell division. (*ii*) Branched differentiation from a common ancestor. (*iii*) Sequential differentiation with obligate cell division; here a certain fraction of the renewal divisions may lead to differentiation into two daughter cells having a new phenotype, e.g., a Tcm dividing into two Tem. (*iv*) A stem-like memory cell may either undergo self-renewal without differentiation or divide asymmetrically to generate one differentiated daughter. (*b*) Describing the fates and persistence of T cell clones. Here we show a hypothetical structure of Tcm and Tem following an immune response, during which both subsets are fed from antigen-stimulated precursors. Each comprises two subpopulations with different rates of division and loss, as was found for CD4 Tem and Tcm in mice (21). On the right we illustrate how the size of each clonal Tcm and Tem population generated early in the response wanes, expressed here as a proportion of the initial clonal T cell population size. In this illustrative example, Tem may be generated either directly from a precursor or through ongoing differentiation from Tcm. The kinetic heterogeneity within each subset gives rise to a biphasic decay of memory clones, as without replenishment fast populations are lost rapidly, leaving the more persistent, slow subpopulation. Abbreviations: Tcm, central memory T cell; Tem, effector memory T cell.

DNA-labeling studies, using either BrdU or deuterium, have shown that in the absence of infection, memory T cells in bulk are kinetically heterogeneous (21, 24, 31). The number of such subpopulations is unclear, and they may even form a continuum; indeed many models of heterogeneity can describe a given labeling time course (32). If one assumes two subpopulations, the data from these studies are consistent with the existence of “fast” cells dividing on a timescale of days and a “slow” population dividing on a timescale of weeks (Figure 2). Such heterogeneity exists within both CD4 Tcm and Tem (15, 21). The relationship between fast and slow memory remains unclear, although it seems plausible that faster populations have recently entered memory and transition to more quiescent populations over time. Potential modes of memory maintenance are shown schematically in Figure 2a, for a hypothetical memory subset comprising two subpopulations with distinct rates of self-renewal and loss.

A challenge for modelers and experimentalists alike is to devise methods of distinguishing these modes of maintenance. For instance, Zarnitsyna et al. (33) observed that the attrition of memory CD8 T cell populations specific for yellow fever virus was best described by power-law kinetics, similar to that described for B cell memory to pertussis (34), although such nonexponential decay curves can be difficult to distinguish from those generated by multiple cell subpopulations with different exponential decay kinetics, leading to a progressive selection for longer-lived memory cells (32, 34).

#### 2.3.2. Cellular dynamics versus clonal dynamics.

For the purposes of sustaining antigen-specific T cell memory, the fates of individual memory cells are perhaps less important than the fates of T cell receptor (TCR) clones themselves. In the very simplest case, if the individual cells comprising a clonal population undergo self-renewal at rate *p* and are lost at rate *d* > *p* (that is, they divide on average every 1*/p* days, and each has an expected life span of 1*/d* days), then the size of that clone decays exponentially, with a clonal life span defined as 1*/*(*d* − *p*). This expression translates to each TCR clone halving in size every log(2)*/*(*d* − *p*) days; if the rates of self-renewal and loss are identical, the clone persists indefinitely. For more complex memory structures, as illustrated in Figure 2b and reviewed in Reference 35, the kinetics of persistence of T cell clones are more difficult to intuit. One must first quantify the rates of division, turnover, and differentiation of each subset, identify lineage relationships, and then simulate the kinetics of survival of each clonotype. Thus, mathematical models are useful tools for predicting how behavior at the single-cell level translates into the key quantity of interest—how functional memory wanes over time—and potentially for suggesting ways to manipulate it.

An instance of such an analysis was the study by Costa Del Amo et al. (36), who explored the idea of stem-like T cell memory cells (Tscm), defined as circulating CD45RA^+^ CD45RO^−^ T cells, as a source of persistent memory clones in humans. They employed in vivo deuterium labeling in humans in an attempt to characterize the dynamic structure of this population. They proposed that Tscm are supplemented at a constant rate from clonally expanded naive T cells, and they fitted models of proliferation, loss, and differentiation to the labeling kinetics in both populations simultaneously. To help isolate the contributions of influx and self-renewal to the maintenance of Tscm numbers, they also used the difference in average lengths of telomeres in the two populations. Data from a cross-sectional study tracking the persistence of CD8 T cell clones specific for an epitope of the yellow fever vaccine were also used to constrain the average clonal life span of the Tscm pool as a whole. They found that the data were consistent with a model in which human CD8 Tscm are comprised of a fast subpopulation with interdivision times of two months and clonal life spans of approximately six months and a slower subset that self-renews rarely and with a clonal life span of more than eight years. It was not possible to define the lineage relationships between these subsets and their naive precursors with certainty, and as Costa Del Amo et al. (36) point out, the existence of a memory population with high degree of self-renewal is compatible with the idea of stemness but is not direct evidence for it. True stem-like behavior, in the sense of a self-renewing precursor capable of dividing asymmetrically to produce one, more differentiated daughter (Figure 2a), is challenging to distinguish from other modes of production with labeling data alone. Doing this likely requires more direct tracking of cell fate with, for example, the cellular bar coding or direct imaging approaches that we discuss below.

### 2.4. Simple Division/Death Models of Cell Dynamics and Differentiation, and Their Limitations

Many successful mathematical models of the dynamics of populations of cells treat division and loss as independent processes. This concept is implied in the models illustrated in Figure 2a. In the simplest models, the probabilities that a cell dies or is triggered to divide within a given time interval are independent of time, of each other, and of the cell’s division history. In statistical terms, division and death are modeled as time-invariant Poisson processes, which imply that cells have no memory of their previous behavior. In these models, the behavior of individual cells is unpredictable, but when cell numbers are sufficiently large, their fluctuations arising from stochastic (random) behavior become relatively small. In this setting we can describe the predicted changes in the total numbers of cells over time with simple, deterministic mathematical models of exponential growth and death, encoded by ordinary differential equations. These models have successfully described the dynamics of naive and memory T cell populations at steady state in many studies of both humans and mice (see Reference 11 for an extensive review; see also Reference 37) and can also capture essential features of immune responses (11, 38, 39).

Models can describe data well, but by their very nature, all of them are wrong at some level; simply, some are less wrong than others. The modeler’s objective is to find an appropriate level of description given a biological question, and the information contained in the data. In some situations, the assumptions underlying a model are clearly at odds with our understanding of cell behavior. For example, the simplest models described above allow cells to divide arbitrarily quickly, in principle. A refinement that explicitly includes the time required to progress through the G1-S-M phases of cell cycle, before returning to a purely probabilistic mode of behavior in quiescence, has also been considered (40–42). This model places a physiological upper limit on the rate that individual cells can divide, but over timescales longer than the cell cycle, cells still have no internal clock or memory of their mitotic history. Further, the process of clonal expansion involves a variable blasting phase of many hours, which varies from cell to cell, followed by an extended burst of deterministic, rapid divisions (43); and in some situations T cells may be at transiently greater risk of death following activation and proliferation (44, 45). These phenomena indicate that in the context of immune responses, we need to broaden our array of models of cellular dynamics and decision making.

## 3. T CELL IMMUNE RESPONSES AND THE FORMATION OF MEMORY AND EFFECTOR CELLS

Mathematical models of the T cell response to a pathogen typically allow for a phase of clonal expansion, during which a population of activated cells divides rapidly, followed by a contraction phase during which most of these cells die, and finally a memory phase during which the surviving population is maintained (11). In some of these models cells are considered to be programmed by the brief initial activation by antigen, because division proceeds even if the antigen is later removed (46). In these “autopilot” models the durations of the expansion and contraction phases are predefined, with distinct rates of proliferation and death rates within each (47–50). Other models have explicit equations for the variation in levels of antigen with time, due to the growth of the pathogen and its subsequent elimination by the immune response, and allow proliferation and death rates to depend on the availability of antigen in the form of peptide-MHC (pMHC) complexes (11, 51, 52). In these models the end of the expansion phase depends on the time at which the pathogen becomes controlled. In both types of models one needs to define how and when cells commit to a memory phenotype (49). Since this was poorly understood, older mathematical models vary considerably in their treatment of the timing and pathways of memory T cell ontogeny. For instance, models have assumed that memory derives from proliferating cells in the expansion phase, or from cells surviving the contraction phase, and have been used to try to distinguish between pathways in which effector T cells convert into memory T cells after antigen clearance and pathways in which memory T cells are formed before differentiation into effector T cells (53). Similar questions have been addressed experimentally, with some studies providing evidence that memory T cells are formed relatively early in the response, before or during the expansion phase (8, 54–58), and another suggesting they arise by dedifferentiation from effector T cells (59). It has also been argued that effector T cells derive from cells that proliferate more extensively during the expansion phase (60, 61) and that, more specifically, differentiation during clonal expansion is linked to the number of divisions the cells have completed (8, 58). These results tell us that to achieve a more mechanistic understanding of the development and diversification of T cell responses, we need to go beyond simple multicompartment models of division, death, and differentiation and develop models for trajectories of individual cells undergoing clonal expansion, linking their fate decisions to their division number (58, 62).

### 3.1. Imprinting of Cell Fate: The Cyton Model

In a series of studies (43, 63–69) Hodgkin and colleagues developed a conceptual framework for describing cell behavior during immune responses. Called the Cyton model (65), it was motivated by the observation that cells responding to antigen appeared to regulate their survival and rates of proliferation independently and that costimulation or cytokine stimuli can modulate both the distributions of the times to first division and the subsequent rates of division and loss (43, 63). In the original formulation of the model, illustrated in figure 1A of Cheon et al. (70), each cell possesses two independent clocks that encode the times that will elapse before it next divides, or dies. Following every division a cell draws a time to die and a time to divide from the two distributions and then undergoes the process destined to be sooner. At the population level, the Cyton model mimics the simple memoryless model of division and death described above if these two distributions are exponential. Yet they encode very different mechanistic descriptions of the cellular machinery: an imprinted fate carried by each cell (Cyton), rather than division and death as purely stochastic processes, akin to radioactive decay, in which cells carry no memory of their previous behavior.

Another defining feature of the Cyton model was that, despite this resetting of these internal clocks after each division, cells inherited a division destiny across generations to explain the observation that the number of divisions undergone during clonal expansion is determined by the combinations of signals received during activation (65, 68). Consistent with the core assumption of the model, the division destiny was shown to be independent of the regulation of the time to die, through manipulation of Bcl-2 expression. Strikingly, the ultimate size of the response could be predicted from the sum of the underlying signal components (68). The Cyton model therefore allows one to encode both quite general rules of T cell behavior in immune responses (times to first division, and distributions of interdivision times) as well as imprinting of cell fate, at the cost of more freedom in its parameterization than is offered by simpler models of cell growth encoded by differential equations. Ultimately the choice of model is usually determined by the level of description or explanatory power one is seeking, and the richness of the data available to fit it to.

### 3.2. Cellular Heterogeneity During Clonal Expansion

Clonal expansion starts with small populations of T cells triggered to proliferate by brief exposures to pMHC and costimulatory signals on the surfaces of activated antigen-presenting cells (46). These cells vary in the number of divisions that they will complete after this initial trigger. This variation is reflected in disparate family sizes (the progeny of each precursor being a family), even among cells that are genetically identical (6, 7, 68, 71, 72). The magnitude of this heterogeneity was revealed directly by in vivo experiments tracing the family sizes of individual progenitor CD8 T cells both by genetic bar coding (7) and with combinations of congenic markers (6). The former study found that after clonal expansion only 5% of the progenitors accounted for the bulk of the immune response overall. Thus, most families divide only a few times and remain small, whereas a few divide many times and dominate the response. Since the magnitude and the timing of the total immune response are quite similar in different mice of the same strain, this reproducibility must be brought about by the summation of hundreds of families of varying sizes.

#### 3.2.1. Sources of heterogeneity in family sizes.

When a family is still composed of just a few cells, any stochasticity in its members’ times to divide and differentiate may lead to large differences in its ultimate size. This “process noise” (73, 74) was sufficient to explain the disparity in family sizes observed in one study (6). This study employed a “memoryless” model (as described above) that was stochastic—that is, one that explicitly modeled the randomness in cell behavior to predict the distribution of cell trajectories, rather than predicting simply the average behavior— and did not assume any inheritance of cell fate (6). Second, genetically identical progenitor cells may differ considerably in the expression levels of various proteins (43, 63) and in the signals they receive upon activation (68), factors that could encode heritable differences in the division rates, death rates, and division destiny of each family (8, 68, 71, 75–77). Direct in vivo evidence for genetically identical OT-1 cells returning to quiescence at different generation numbers during clonal expansion was obtained in FucciRG mice, where cessation of division results in cells reacquiring red fluorescence (68). The observed distribution of division destinies depended on the affinity of the cells and the availability of IL-2, which was all well described by fitting a Cyton model (68). Indeed, the Cyton model was originally developed with the ideas that every cell is different and that simple stochastic processes sampling from similar distributions for the times to the next division, to die, or to stop dividing can yield substantial variation in family sizes and combine to generate a reproducible total immune response (78). One of the factors defining these parameters within a family is the expression of Myc, whose base production rate is set by signals received upon activation and then declines at a universal rate. Cells stop dividing when Myc expression falls below a critical threshold, suggesting that a cell’s division destiny is set by a timer rather than a division counter (69, 79). The fact that division and death rates are correlated between daughter and mother cells (8, 77) and the fact that in many families cells stop dividing around the same division number (71) suggest that mechanistic mathematical models for clonal expansion should be written as a sum of many families, each having its own parameters. Third, the local environment in which a family is expanding will be markedly different in vivo (80, 81), and at least somewhat different in vitro (8), probably translating into intrinsically different parameters for each individual family. Due to positive feedback where rapidly expanding families better recruit additional antigen-presenting cells (81), small initial differences in the local environment may readily lead to large differences in family sizes.

**Figure 3:**
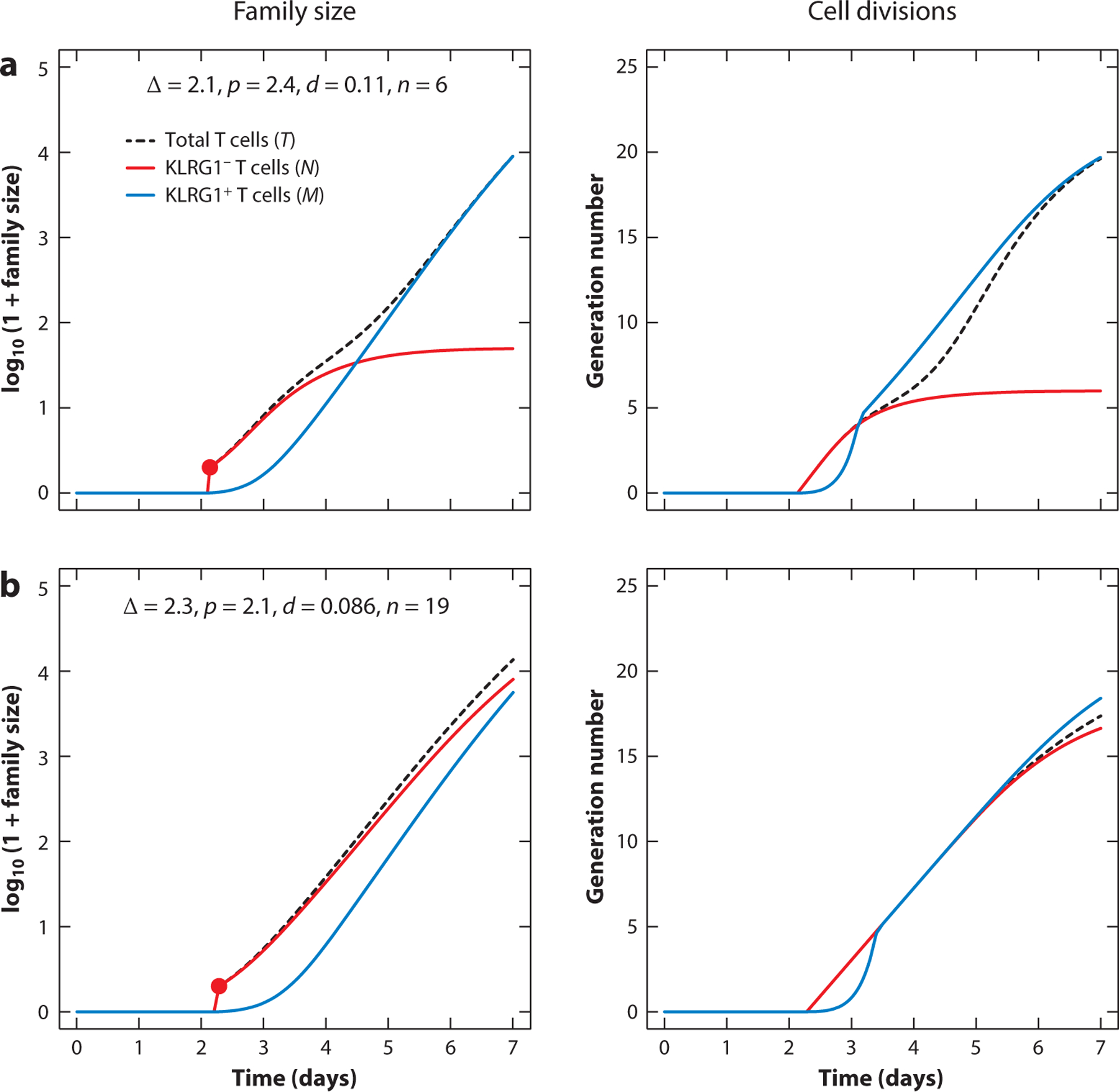
Modeling heterogeneity in T cell family dynamics. Following the model of Bresser et al. (58), consider a family that proliferates and differentiates during a seven-day clonal expansion phase. The family starts with one KLRG1^−^ cell, *N*_0_ (indicated by the red circle), that begins to divide and differentiate after a delay, Δ. Subsequently, the number of cells in each generation is determined by the equations d*N*_*i*_*/*d*t* = 2*p*_*i −* 1_*N*_*i –* 1 −_(*p*_*i*_ + *d*_*i*_)*N*_*i*_, where the division rate, *p*_*i*_, and the differentiation rate, *d*_*i*_, both may depend on the number of divisions, *i*, the cells have completed. These activated cells are assumed to become quiescent after a certain number of divisions *n*; i.e., *p*_*i* =_ *d*_*i* =_ 0 when *I* ≥ *n*. Nonquiescent proliferating cells can differentiate into KLRG1^+^ cells that continue to divide (at the same rate *p*_*i*_, for simplicity); i.e., d*M*_*i*_*/*d*t = d*_*i*_*N*_*i*_ + 2*p*_*i −* 1_*M*_*i –* 1 −_ *p*_*i*_*M*_*i*_. A typical immune response comprises hundreds of these families, each having unique parameter values. We depict two examples, with the first family (*a*) returning to quiescence at division *n* = 6, and the second (*b*) at *n* = 19. The panels on the left depict the size of a family stratified by phenotype, *N = ∑*_*i*_ *N*_*i*_ and *M* = ∑*i M*_*i*_, and its total size, *T* = *N* + *M*. The panels on the right depict the average generation number of each phenotype, e.g., *g* = ∑_*i*_ *i N*_*i*_*/∑i N*_*i*_ (82). For simplicity, the division and death rates do not depend on the generation number in these two simulations: We set them to *p*_*i*_ = *p* and *d*_*i* =_ *d* and indicate these values in the panels on the left. (Here, 1*/p* is the mean interdivision time in days, and 1*/d* is the average time it takes cells within the dividing population to become KLRG1^+^.)

The division-indexed model developed by Bresser et al. (58) can be used to illustrate the dynamics of individual families. The model explained in the legend of Figure 3 depicts one week of clonal expansion of a family founded by a single precursor cell that starts to divide after a time that depends on its local environment and/or levels of antigen and costimulation. This delay typically follows a log-normal (bell-shaped) distribution (7, 63, 70). To model family-based division destiny (71), Bresser et al. (58) assumed that nondifferentiated cells in a family could return to quiescence after a particular number of divisions, in a probabilistic manner. Proliferating cells can differentiate by expressing KLRG1 and then continuing to proliferate during the clonal expansion phase, without reverting to quiescence. The total immune response is the sum of many families (Figure 3 depicts just two examples), each having unique proliferation and differentiation rates, a unique time to first division, and a unique division destiny (see the legend of Figure 3). Because this model is based upon differential equations, its predictions are smooth curves that represent the expected size of the family comprised of cells each with exponentially distributed times to divide and die—essentially, the average family size that would be observed over repeated stimulations with exactly the same underlying parameters.

Family-based models such as this one, even without the differentiation component (7), naturally explain the heterogeneity observed in the contribution of each family to the total immune response. The predicted disparity would be even greater if larger families recruit additional activated antigen-presenting cells to their local environment more rapidly than those that start later and proliferate more slowly (81), i.e., if there is an acceleration of the proliferation rate within families. To describe the dynamics of growth and differentiation within families in more detail, Pandit & De Boer (62) developed a model that assumed that division and death times were inherited by cells’ offspring, with some noise associated (62). By describing population growth and differentiation as a type of stochastic model known as a branching process, this model was able to predict both the mean and the spread of possible outcomes and could explain both the family size disparity and the distributions of CD62L, CD27, and KLRG1 expression reported by Buchholz et al. (6). Family-based models have additional important properties that are not captured by models of the total immune response. For instance, families starting early and proliferating rapidly will progressively dominate, which implies that during the clonal expansion phase the average proliferation rate of the entire immune response will increase over time (62). The latter was indeed observed when the data of Buchholz et al. were fitted with a model in which cells differentiate sequentially into multiple effector and memory phenotypes (6). Additionally, one requires large differences in the number of generations within families to explain the observation of Bresser et al. (58) that responses to secondary or tertiary challenges with *Listeria monocytogenes* are seeded with memory cells having a low generation number.

### 3.3. Heritable Properties Within T Cell Families

Filming the clonal expansion of individual B and T cells in vitro over several generations provided strong evidence that the time to complete the first division, the subsequent division and death rates, and the division destiny differ considerably between families, and are more similar within families, as a consequence of correlations between daughter and mother cells (8, 70, 75, 77). Prompted by novel long-term live imaging of proliferating CD8 T cells (70), and the recent accumulation of the evidence obtained in vivo (6, 7, 68) and in vitro (68, 69, 71) confirming that families differ considerably in their clonal expansion, kinetics, and division destiny, Cheon et al. (70) revised the original Cyton model. In Cyton2 (see figure 1B in Reference 70), all cells within a family are destined to die at the same point in time (71), members of the same generation have the same interdivision time (70), and cells continue to divide for a time corresponding to the division destiny or their time of death, whichever is sooner (69). Thus, the new Cyton2 model uses random variables to define for each family a time to first division, a global death timer, a global destiny timer, and an interdivision time that is the same within generations but may be different across generations. Thanks to this fairly simple structure, with a limited number of parameters within each family, the Cyton2 model could be fitted to the data obtained from the imaging; that is, Cheon et al. (70) could identify the forms of the distributions followed by these parameters. This was confirmed by simulations with an agent-based model in which each cell is modeled individually and behaves probabilistically according to the rules of the Cyton2 model. Their new model was also able to describe the linear integration of costimulatory signals received by precursor cells observed previously (68).

As yet, the Cyton2 model lacks mechanisms for differentiation into memory and effector precursors (58), but novel filming experiments are already providing clues to how these could be implemented. By continuous filming of clonally expanding CD8 T cells in macrowells in which cells can migrate freely, a recent study managed to track the development individual families up to ten generations in size, over five days (8). A Bayesian inference framework was developed to fit several mechanistically different branching process models to the genealogical trees obtained from these movies, each model assuming log-normally distributed interdivision times. The branching process models differed in the number of subsets with different proliferation rates allowed within each family. In the model with strongest support, (*a*) the division rate was unique during the first few generations, (*b*) later generations had slowly and rapidly dividing branches, and (*c*) each family had its own division rate. This model confirmed that the heterogeneity in kinetics across CD8 T cell families is much larger than that within families. Plambeck et al. (8) also concluded that in most families completion of the first division took more than a day (39 h on average), and that cells then rapidly completed two or three divisions (mean 8.6 h per division). This behavior mirrors the very rapid initial proliferation rate of CD8 T cells stimulated in vivo with influenza (83, 84). In the absence of further TCR stimulation, the division rate slowed, resembling the division destiny encoded in the Cyton model (68, 70, 71). However with continuous TCR stimulation the division rate remained high, with cells segregating into distinct branches of slowly and rapidly dividing cells (8). The division rate during the initial burst of divisions was independent of levels of exogenous IL-2 and IL-12, but expression of the IL-2 receptor α chain (CD25) during the burst phase was correlated with the subsequent rate of cell division. Moreover, the slowly dividing branches typically expressed higher levels of CD62L, suggesting that these were predestined to form memory cells. These data confirm that the decision to commit to a less proliferative memory phenotype is made during the early stages of clonal expansion (54–58). It remains puzzling that despite the relation between division rates and fate observed by Plambeck et al. (8) in vitro, recent studies in vivo have demonstrated that memory and effector precursors have completed a similar number of divisions, on average, at the end of the clonal expansion phase (58, 85).

### 3.4. Understanding the Relationship Between Memory Formation and Division History

In an acute viral infection, there is a contraction phase following clonal expansion in which approximately 95% of the responding CD8 T cells die, with the surviving 5% forming a persistent population of memory cells (49). This population is phenotypically diverse, including Tcm, Tem, and Tscm (85, 86). As described above, several conceptual and mathematical models of the timing and pathways of development of these populations have been proposed (6, 49, 53, 59, 86). The emergence of new tools for mapping the diversification within T cell responses allows us to revisit these controversies.

A recent development was to engineer a division recorder into progenitor CD8 T cells and transfer these cells to mice that were then challenged with *L. monocytogenes*, to study the proliferative history of memory populations (58). The division recorder system was proposed by Weber et al. (87), who used mathematical modeling to demonstrate that a genetic construct that changes upon cell division, with a sufficiently low probability, would provide a readout of the number of divisions the population has completed. This proposal was realized in a system in which TCR transgenic OT-1 T cells have an out-of-frame Cre recombinase gene and an RFP gene located after a stop codon. Rare slippage mutations during cell division allow Cre to become expressed, which excises the stop codon, allowing RFP to become permanently expressed and inherited by that cell’s progeny (58). Thus, with a small probability a dividing cell delivers a family of descendants that are indelibly fluorescent. Importantly, for sufficiently low slippage probabilities, the fraction of cells expressing RFP is proportional to the average generation number of the population and is independent of the death rate of the cells (58, 87).

Bresser et al. (58) recorded time courses of the numbers of CD27^+^KLRG1^−^ Tcm (or Tcm precursor) and CD27^−^KLRG1^+^ Tem (or Tem precursor) populations, and their redness (the fraction of each expressing RFP). They showed that at the peak of the response, around day 6, the Tcm and Tem precursor populations both accumulated about 10 divisions on average. Thus, in their system, memory is formed from cells that have divided extensively. During the subsequent memory phase the Tcm continued to divide (became redder), accumulating another 20 divisions by day 86, whereas the Tem population showed little increase in redness (58). The latter observation shows that during the memory phase, Tem hardly divide and that Tcm differentiate into Tem only at very low levels, if at all. To study which memory cells are responsible for mounting responses to repeated exposures, the mice were rechallenged with *Listeria* late in the memory phase. Surprisingly, the RFP^+^ fractions among Tcm and Tem declined markedly during the first few days of a secondary and tertiary immune response, indicating that both were replenished from a persisting population of quiescent memory cells that had completed relatively few divisions.

Mathematical modeling confirmed this interpretation and provided estimates of the minimum number of quiescent cells and the maximum number of divisions these cells must have completed in order to explain a large drop in the RFP^+^ fraction (58). Thus, protection against secondary or tertiary challenge requires an immune response dominated by memory cells (or families) that previously stopped clonal expansion early. (The first family in Figure 3 would be an example, as the undifferentiated cells accumulate only six to seven divisions, whereas the second family accumulates almost as many divisions as the differentiated cells.) If such cells were maintained quiescently during the subsequent contraction and memory phase, they could readily account for large expansion following another challenge. Note that during that response, a small subset of the families will again stop early and form new populations of quiescent memory T cells (58).

These data were interpreted using a division-indexed mathematical model like the one described in the legend of Figure 3, where its two populations correspond to Tcm and Tem. The model of Bresser et al. (58) is more complicated, as it also tracks the red subpopulation of both phenotypes, and also defined a contraction phase, a memory phase, and another expansion phase to model the secondary challenge. The simplified model is similar to the models developed by Schlub et al. (88), who proposed that Tcm can differentiate to Tem with a certain probability per division, which also allows Tem to emerge at high division numbers. An adequate description of the data required that sufficient quiescent cells are formed early in the response and that during clonal expansion the rate of differentiation into CD27^−^KLRG1^+^ cells increases over time. To preferentially recruit quiescent memory cells into the secondary response, the model assumed that the probability of recruitment declined with the cell’s division number (58). Thus, Tcm that had divided many times were much less likely to proliferate after rechallenge and were rather assumed to leave the circulation to provide immediate protection. Because the model was not family based, quiescent cells were assumed to be formed continuously by branching off during clonal expansion, and not en masse from families stopping division early (58). Thus the explanatory power of this model was directed at understanding broadly the effect of cells’ division histories on their participation in immune responses, rather than explaining their detailed clonal (familial) structure.

#### 3.4.1. Division history also impacts memory in the setting of chronic infections.

The conclusions of Bresser et al. (58) are consistent with another recent analysis of memory T cell ontogeny and maintenance. Chronic cytomegalovirus infections are known to drive large clonal expansions of CD8 T cells, in both mice and humans (89, 90). Using a murine cytomegalovirus (MCMV) model, Grassmann et al. (72) found that families of cells that underwent fewer divisions during the acute response came to dominate memory during the chronic phase of infection. These cells exhibited a Tcm-like phenotype, with reduced effector function and expression of TCF-1, a transcription factor associated with memory maintenance (91–93). Grassmann et al. concluded that persistent memory arose from cells that committed to a central memory phenotype relatively early in the response. Notably, they used a TCR transgenic system, with OT-1 CD8 T cells responding to MCMV engineered to express an OVA epitope. Only 10% of responding monoclonal populations formed these small families, which were later overrepresented in memory, indicating that TCR affinity alone is not the sole determinant of the degree of expansion, and reaffirming that stochasticity in pMHC availability, costimulation, cytokine milieu, and/or gene expression also influences the fate of families. The observed sensitivity of the overall size of the CD8 T cell response to the dose of yellow fever virus (94) may also reflect the influence of peptide availability on the distribution of family sizes. Grassmann et al. also found that the inflated Tcm OT-1 cells isolated from the chronically infected mice were much better able to generate inflated populations following adoptive transfer and challenge than Tem or effector cells. A concurrent study demonstrated that stochastic bursts of proliferation of CD8^+^ memory cells underlie the persistence of the clone hierarchy in the inflated phase of chronic MCMV infection and suggested that these bursts derive from less-differentiated populations (95).

#### 3.4.2. Extending family-based models.

The detailed models of the family dynamics reviewed here do not account yet for the observations by Plambeck et al. (8) that activated cells initially divide rapidly before segregating into phenotypes with different division rates. It also remains unclear how the quiescent cells responsible for subsequent immune responses fit into this picture, and why the CD27^+^KLRG1^−^ (Tcm-like) and CD27^−^KLRG1^+^ (Tem-like) phenotypes at the peak of the response have completed a similar number of divisions in the data of Bresser et al. (58). Models require refinement to explain these data.

In summary, the richness of recent data has revealed that to model the formation of CD8 T cell memory in detail, at a clonal level, one needs to invoke family-based, division-linked models to allow (*a*) some families to stop early and form quiescent memory T cells, (*b*) some families to divide faster than others, and (*c*) the rate of differentiation into subsequent phenotypes to be linked to the number of divisions cells have completed.

## 4. CONCLUSION

This review has focused on the fates and decisions of mature naive and antigen-experienced T cells. Multiple, complex fate decisions are made during the development of T cell subsets in the thymus, which we have not discussed here. Modeling approaches to understanding these processes have been reviewed elsewhere (96).

We have reviewed several mathematical models describing the clonal expansion of CD8 T cells during an immune response. We have argued that to best describe cellular differentiation and the heterogeneous contributions of individual naive precursors in a mechanistic manner, these models should be descriptive at the level of individual families and allow for the rates (or probabilities) of division, loss, and differentiation to depend on generation number. Not all models describing clonal expansion need to be that complicated, however; in general, their complexity should depend on the question being addressed. Whenever one is aiming to describe a typical immune response, e.g., to quantify parameters defining its timing and magnitude, it is often more appropriate to use simplified, more phenomenological models with many fewer parameters. Indeed the many unknown parameters of the family-based generation-indexed models are not identifiable from the fairly limited time course data that we typically collect from in vivo immune responses. However, the detailed mechanistic models help us to better define these simpler averaged models because they provide information regarding the timing of differentiation processes, and how much variance in the overall timing and magnitude of the immune response one might expect.

The diversification of T cell responses is also linked to their location, as responding cells migrate from lymphoid organs to other tissues, where they undergo site-specific adaptations (97–99). Preserving spatial information when studying antigen-specific T cell populations may therefore help to impose some order on their heterogeneity and allow us to distinguish between candidate developmental pathways. At the same time, extending these mathematical models to describe the dynamics of T cells in space as well as time increases the number of parameters that must be estimated. The challenge is to find the optimal level of description in which just enough spatial information is included in our models to make them both useful and tractable.

A common problem when comparing and synthesizing studies of T cell memory is a lack of consensus on the finer-grained phenotypic definitions of antigen-experienced T cell subsets. This issue arises also with unbiased approaches to defining discrete phenotypes, either with single-cell sequencing data or high-dimensional flow cytometry, which can exhibit strong batch effects, even across replicate experiments. However, it seems that despite this lack of consensus, at the level of canonical surface markers we are beginning to develop a consistent conceptual picture of how heterogeneity and memory emerge from T cell responses. Perhaps the next challenge is to characterize the dynamics of maintenance of T cell memory long-term, which may bring us closer to answering the long-standing question of what determines the longevity of T cell memory to pathogen epitopes. As increasingly powerful experimental tools become available to us, judicious use of mathematical models may become essential for interpreting the data they produce.

## References

[1] Kaech SM, Wherry EJ. 2007. Heterogeneity and cell-fate decisions in effector and memory CD8^+^ T cell differentiation during viral infection. Immunity 27(3):393–405 17892848 10.1016/j.immuni.2007.08.007 PMC3431921

[2] Ahmed R, Bevan MJ, Reiner SL, Fearon DT. 2009. The precursors of memory: models and controversies. Nat. Rev. Immunol 9(9):662–68 19680250 10.1038/nri2619

[3] Chang JT, Wherry EJ, Goldrath AW. 2014. Molecular regulation of effector and memory T cell differentiation. Nat. Immunol 15(12):1104–15 25396352 10.1038/ni.3031 PMC4386685

[4] Jameson SC, Masopust D. 2018. Understanding subset diversity in T cell memory. Immunity 48(2):214–26 29466754 10.1016/j.immuni.2018.02.010 PMC5863745

[5] Bird JJ, Brown DR, Mullen AC, Moskowitz NH, Mahowald MA, 1998. Helper T cell differentiation is controlled by the cell cycle. Immunity 9(2):229–37 9729043 10.1016/s1074-7613(00)80605-6

[6] Buchholz VR, Flossdorf M, Hensel I, Kretschmer L, Weissbrich B, 2013. Disparate individual fates compose robust CD8^+^ T cell immunity. Science 340(6132):630–35 23493420 10.1126/science.1235454

[7] Gerlach C, Rohr JC, Perié L, van Rooij N, van Heijst JWJ, 2013. Heterogeneous differentiation patterns of individual CD8^+^ T cells. Science 340(6132):635–39 23493421 10.1126/science.1235487

[8] Plambeck M, Kazeroonian A, Loeffler D, Kretschmer L, Salinno C, 2022. Heritable changes in division speed accompany the diversification of single T cell fate. PNAS 119(9):e2116260119 35217611 10.1073/pnas.2116260119 PMC8892279

[9] Stemberger C, Huster K, Koffler M, Anderl F, Schiemann M, 2007. A single naive CD8^+^ T cell precursor can develop into diverse effector and memory subsets. Immunity 27(6):985–97 18082432 10.1016/j.immuni.2007.10.012

[10] Buchholz VR, Schumacher TNM, Busch DH. 2016. T cell fate at the single-cell level. Annu. Rev. Immunol 34:65–92 26666651 10.1146/annurev-immunol-032414-112014

[11] De Boer RJ, Perelson AS. 2013. Quantifying T lymphocyte turnover. J. Theor. Biol 327:45–87 23313150 10.1016/j.jtbi.2012.12.025 PMC3640348

[12] Hogan T, Yates A, Seddon B. 2017. Generation of busulfan chimeric mice for the analysis of T cell population dynamics. Bio-protocol 4(24):e2650 10.21769/BioProtoc.2650 PMC8438465 34595313

[13] Hogan T, Gossel G, Yates AJ, Seddon B. 2015. Temporal fate mapping reveals age-linked heterogeneity in naive T lymphocytes in mice. PNAS 112(50):E6917–26 26607449 10.1073/pnas.1517246112 PMC4687551

[14] Asquith B, Debacq C, Macallan DC, Willems L, Bangham CR. 2002. Lymphocyte kinetics: the interpretation of labelling data. Trends Immunol. 23(12):596–601 12464572 10.1016/s1471-4906(02)02337-2

[15] Hogan T, Nowicka M, Cownden D, Pearson CF, Yates AJ, Seddon B. 2019. Differential impact of self and environmental antigens on the ontogeny and maintenance of CD4^+^ T cell memory. eLife 8:e48901 31742553 10.7554/eLife.48901 PMC6905650

[16] Rane S, Hogan T, Seddon B, Yates AJ. 2018. Age is not just a number: Naive T cells increase their ability to persist in the circulation over time. PLOS Biol. 16(4):e2003949 29641514 10.1371/journal.pbio.2003949 PMC5894957

[17] Rane S, Hogan T, Lee E, Seddon B, Yates AJ. 2022. Towards a unified model of naive T cell dynamics across the lifespan. eLife 11:e78168 35678373 10.7554/eLife.78168 PMC9348855

[18] Tsukamoto H, Clise-Dwyer K, Huston GE, Duso DK, Buck AL, 2009. Age-associated increase in lifespan of naive CD4 T cells contributes to T-cell homeostasis but facilitates development of functional defects. PNAS 106(43):18333–38 19815516 10.1073/pnas.0910139106 PMC2759371

[19] Houston EG Jr., Higdon LE, Fink PJ. 2011. Recent thymic emigrants are preferentially incorporated only into the depleted T-cell pool. PNAS 108(13):5366–71 21402911 10.1073/pnas.1015286108 PMC3069187

[20] den Braber I, Mugwagwa T, Vrisekoop N, Westera L, Mögling R, 2012. Maintenance of peripheral naive T cells is sustained by thymus output in mice but not humans. Immunity 36(2):288–97 22365666 10.1016/j.immuni.2012.02.006

[21] Gossel G, Hogan T, Cownden D, Seddon B, Yates AJ. 2017. Memory CD4 T cell subsets are kinetically heterogeneous and replenished from naive T cells at high levels. eLife 6:e23013 28282024 10.7554/eLife.23013 PMC5426903

[22] Kawabe T, Jankovic D, Kawabe S, Huang Y, Lee PH, 2017. Memory-phenotype CD4^+^ T cells spontaneously generated under steady-state conditions exert innate T_H_1-like effector function. Sci. Immunol 2(12):eaam9304 28783663 10.1126/sciimmunol.aam9304 PMC5568832

[23] van Hoeven V, Drylewicz J, Westera L, den Braber I, Mugwagwa T, 2017. Dynamics of recent thymic emigrants in young adult mice. Front. Immunol 8:933 28824653 10.3389/fimmu.2017.00933 PMC5545745

[24] Younes SA, Punkosdy G, Caucheteux S, Chen T, Grossman Z, Paul WE. 2011. Memory phenotype CD4 T cells undergoing rapid, nonburst-like, cytokine-driven proliferation can be distinguished from antigen-experienced memory cells. PLOS Biol. 9(10):e1001171 22022231 10.1371/journal.pbio.1001171 PMC3191130

[25] Aghajani K, Keerthivasan S, Yu Y, Gounari F. 2012. Generation of CD4CreER^T2^ transgenic mice to study development of peripheral CD4-T-cells. Genesis 50(12):908–13 22887772 10.1002/dvg.22052 PMC3535561

[26] Reynaldi A, Smith NL, Schlub TE, Tabilas C, Venturi V, 2019. Fate mapping reveals the age structure of the peripheral T cell compartment. PNAS 116(10):3974–81 30765525 10.1073/pnas.1811634116 PMC6410819

[27] Smith NL, Patel RK, Reynaldi A, Grenier JK, Wang J, 2018. Developmental origin governs CD8^+^ T cell fate decisions during infection. Cell 174(1):117–30.e14 29909981 10.1016/j.cell.2018.05.029

[28] Macallan DC, Borghans JAM, Asquith B. 2017. Human T cell memory: a dynamic view. Vaccines 5(1):5 28165397 10.3390/vaccines5010005 PMC5371741

[29] Fearon DT, Manders P, Wagner SD. 2001. Arrested differentiation, the self-renewing memory lymphocyte, and vaccination. Science 293(5528):248–50 11452114 10.1126/science.1062589

[30] Gattinoni L, Zhong XS, Palmer DC, Ji Y, Hinrichs CS, 2009. Wnt signaling arrests effector T cell differentiation and generates CD8^+^ memory stem cells. Nat. Med 15(7):808–13 19525962 10.1038/nm.1982 PMC2707501

[31] Westera L, Drylewicz J, den Braber I, Mugwagwa T, van der Maas I, 2013. Closing the gap between T-cell life span estimates from stable isotope-labeling studies in mice and humans. Blood 122(13):2205–12 23945154 10.1182/blood-2013-03-488411

[32] Ganusov VV, Borghans JAM, De Boer RJ. 2010. Explicit kinetic heterogeneity: mathematical models for interpretation of deuterium labeling of heterogeneous cell populations. PLOS Comput. Biol 6(2):e1000666 20140186 10.1371/journal.pcbi.1000666 PMC2816685

[33] Zarnitsyna VI, Akondy RS, Ahmed H, McGuire DJ, Zarnitsyn VG, 2021. Dynamics and turnover of memory CD8 T cell responses following yellow fever vaccination. PLOS Comput. Biol 17(10):e1009468 34648489 10.1371/journal.pcbi.1009468 PMC8568194

[34] Teunis PFM, van Eijkeren JCH, de Graaf WF, Marinović AB, Kretzschmar MEE. 2016. Linking the seroresponse to infection to within-host heterogeneity in antibody production. Epidemics 16:33–39 27663789 10.1016/j.epidem.2016.04.001

[35] Restifo NP, Gattinoni L. 2013. Lineage relationship of effector and memory T cells. Curr. Opin. Immunol 25(5):556–63 24148236 10.1016/j.coi.2013.09.003 PMC3858177

[36] Costa Del Amo P, Beneytez JL, Boelen L, Ahmed R, Miners KL, 2018. Human T_SCM_ cell dynamics in vivo are compatible with long-lived immunological memory and stemness. PLOS Biol. 16(6):1–22 10.1371/journal.pbio.2005523 PMC6033534 29933397

[37] Costa Del Amo P, Debebe B, Razavi-Mohseni M, Nakaoka S, Worth A, 2020. The rules of human T cell fate in vivo. Front. Immunol 11:573 32322253 10.3389/fimmu.2020.00573 PMC7156550

[38] Perelson AS. 2002. Modelling viral and immune system dynamics. Nat. Rev. Immunol 2(1):28–36 11905835 10.1038/nri700

[39] Perelson AS, Ribeiro RM. 2013. Modeling the within-host dynamics of HIV infection. BMC Biol. 11:96 24020860 10.1186/1741-7007-11-96 PMC3765939

[40] De Boer RJ, Ganusov VV, Milutinovic D, Hodgkin PD, Perelson AS. 2006. Estimating lymphocyte division and death rates from CFSE data. Bull. Math. Biol 68(5):1011–31 16832737 10.1007/s11538-006-9094-8

[41] Ganusov VV, Pilyugin SS, de Boer RJ, Murali-Krishna K, Ahmed R, Antia R. 2005. Quantifying cell turnover using CFSE data. J. Immunol. Methods 298(1–2):183–200 15847808 10.1016/j.jim.2005.01.011

[42] Smith JA, Martin L. 1973. Do cells cycle? PNAS 70(4):1263–67 4515625 10.1073/pnas.70.4.1263 PMC433472

[43] Gett AV, Hodgkin PD. 2000. A cellular calculus for signal integration by T cells. Nat. Immunol 1(3):239–44 10973282 10.1038/79782

[44] Yates A, Stark J, Klein N, Antia R, Callard R. 2007. Understanding the slow depletion of memory CD4^+^ T cells in HIV infection. PLOS Med. 4(5):e177 17518516 10.1371/journal.pmed.0040177 PMC1872038

[45] Nolz JC, Rai D, Badovinac VP, Harty JT. 2012. Division-linked generation of death-intermediates regulates the numerical stability of memory CD8 T cells. PNAS 109(16):6199–204 22474367 10.1073/pnas.1118868109 PMC3341021

[46] Van Stipdonk MJ, Lemmens EE, Schoenberger SP. 2001. Naive CTLs require a single brief period of antigenic stimulation for clonal expansion and differentiation. Nat. Immunol 2:423–29 11323696 10.1038/87730

[47] De Boer RJ, Oprea M, Antia R, Murali-Krishna K, Ahmed R, Perelson AS. 2001. Recruitment times, proliferation, and apoptosis rates during the CD8^+^ T-cell response to lymphocytic choriomeningitis virus. J. Virol 75:10663–69 11602708 10.1128/JVI.75.22.10663-10669.2001 PMC114648

[48] De Boer RJ, Homann D, Perelson AS. 2003. Different dynamics of CD4^+^ and CD8^+^ T cell responses during and after acute lymphocytic choriomeningitis virus infection. J. Immunol 171:3928–35 14530309 10.4049/jimmunol.171.8.3928

[49] Antia R, Ganusov VV, Ahmed R. 2005. The role of models in understanding CD8^+^ T-cell memory. Nat. Rev. Immunol 5(2):101–11 15662368 10.1038/nri1550

[50] Kohler B 2007. Mathematically modeling dynamics of T cell responses: predictions concerning the generation of memory cells. J. Theor. Biol 245(4):669–76 17222868 10.1016/j.jtbi.2006.10.017

[51] Antia R, Bergstrom CT, Pilyugin SS, Kaech SM, Ahmed R. 2003. Models of CD8^+^ responses: 1. What is the antigen-independent proliferation program. J. Theor. Biol 221(4):585–98 12713942 10.1006/jtbi.2003.3208

[52] De Boer RJ, Perelson AS. 2013. Antigen-stimulated CD4 T cell expansion can be limited by their grazing of peptide-MHC complexes. J. Immunol 190(11):5454–58 23606541 10.4049/jimmunol.1203569 PMC3661195

[53] Ganusov VV, Milutinovic D, De Boer RJ. 2007. IL-2 regulates expansion of CD4^+^ T cell populations by affecting cell death: insights from modeling CFSE data. J. Immunol 179(2):950–57 17617586 10.4049/jimmunol.179.2.950

[54] Joshi NS, Cui W, Chandele A, Lee HK, Urso DR, 2007. Inflammation directs memory precursor and short-lived effector CD8^+^ T cell fates via the graded expression of T-bet transcription factor. Immunity 27(2):281–95 17723218 10.1016/j.immuni.2007.07.010 PMC2034442

[55] Pais Ferreira D, Silva JG, Wyss T, Fuertes Marraco SA, Scarpellino L, 2020. Central memory CD8^+^ T cells derive from stem-like Tcf7^hi^ effector cells in the absence of cytotoxic differentiation. Immunity 53(5):985–1000 33128876 10.1016/j.immuni.2020.09.005

[56] Kretschmer L, Flossdorf M, Mir J, Cho YL, Plambeck M, 2020. Differential expansion of T central memory precursor and effector subsets is regulated by division speed. Nat. Commun 11(1):113 31913278 10.1038/s41467-019-13788-w PMC6949285

[57] Johnnidis JB, Muroyama Y, Ngiow SF, Chen Z, Manne S, 2021. Inhibitory signaling sustains a distinct early memory CD8^+^ T cell precursor that is resistant to DNA damage. Sci. Immunol 6(55):1563–73 10.1126/sciimmunol.abe3702 PMC8258400 33452106

[58] Bresser K, Kok L, Swain AC, King LA, Jacobs L, 2022. Replicative history marks transcriptional and functional disparity in the CD8^+^ T cell memory pool. Nat. Immunol 23(5):791–801 35393592 10.1038/s41590-022-01171-9 PMC7612726

[59] Youngblood B, Hale JS, Kissick HT, Ahn E, Xu X, 2017. Effector CD8 T cells dedifferentiate into long-lived memory cells. Nature 552(7685):404–9 29236683 10.1038/nature25144 PMC5965677

[60] Sarkar S, Kalia V, Haining WN, Konieczny BT, Subramaniam S, Ahmed R. 2008. Functional and genomic profiling of effector CD8 T cell subsets with distinct memory fates. J. Exp. Med 205(3):625–40 18316415 10.1084/jem.20071641 PMC2275385

[61] Kalia V, Sarkar S, Subramaniam S, Haining WN, Smith KA, Ahmed R. 2010. Prolonged interleukin-2Rα expression on virus-specific CD8^+^ T cells favors terminal-effector differentiation in vivo. Immunity 32(1):91–103 20096608 10.1016/j.immuni.2009.11.010

[62] Pandit A, De Boer RJ. 2019. Stochastic inheritance of division and death times determines the size and phenotype of CD8^+^ T cell families. Front. Immunol 10:436 30923522 10.3389/fimmu.2019.00436 PMC6426761

[63] Deenick EK, Gett AV, Hodgkin PD. 2003. Stochastic model of T cell proliferation: a calculus revealing IL-2 Regulation of precursor frequencies, cell cycle time, and survival. J. Immunol 170(10):4963–72 12734339 10.4049/jimmunol.170.10.4963

[64] Dowling MR, Milutinović D, Hodgkin PD. 2005. Modelling cell lifespan and proliferation: Is likelihood to die or to divide independent of age? J. R. Soc. Interface 2(5):517–26 16849210 10.1098/rsif.2005.0069 PMC1618504

[65] Hawkins ED, Turner ML, Dowling MR, van Gend C, Hodgkin PD. 2007. A model of immune regulation as a consequence of randomized lymphocyte division and death times. PNAS 104(12):5032–37 17360353 10.1073/pnas.0700026104 PMC1821128

[66] Dowling MR, Hodgkin PD. 2009. Modelling naive T-cell homeostasis: consequences of heritable cellular lifespan during ageing. Immunol. Cell Biol 87(6):445–56 19290017 10.1038/icb.2009.11

[67] Duffy KR, Hodgkin PD. 2012. Intracellular competition for fates in the immune system. Trends Cell Biol. 22(9):457–64 22727035 10.1016/j.tcb.2012.05.004

[68] Marchingo JM, Kan A, Sutherland RM, Duffy KR, Wellard CJ, 2014. Antigen affinity, costimulation, and cytokine inputs sum linearly to amplify T cell expansion. Science 346(6213):1123–27 25430770 10.1126/science.1260044

[69] Heinzel S, Binh Giang T, Kan A, Marchingo JM, Lye BK, 2017. A Myc-dependent division timer complements a cell-death timer to regulate T cell and B cell responses. Nat. Immunol 18(1):96–103 27820810 10.1038/ni.3598

[70] Cheon H, Kan A, Prevedello G, Oostindie SC, Dovedi SJ, 2021. Cyton2: a model of immune cell population dynamics that includes familial instructional inheritance. Front. Bioinformat 1:723337 10.3389/fbinf.2021.723337 PMC9581048 36303793

[71] Marchingo JM, Prevedello G, Kan A, Heinzel S, Hodgkin PD, Duffy KR. 2016. T-cell stimuli independently sum to regulate an inherited clonal division fate. Nat. Commun 7:13540 27869196 10.1038/ncomms13540 PMC5121331

[72] Grassmann S, Mihatsch L, Mir J, Kazeroonian A, Rahimi R, 2020. Early emergence of T central memory precursors programs clonal dominance during chronic viral infection. Nat. Immunol 21(12):1563–73 33106669 10.1038/s41590-020-00807-y

[73] Milutinović D, De Boer RJ. 2007. Process noise: an explanation for the fluctuations in the immune response during acute viral infection. Biophys. J 92(10):3358–67 17325020 10.1529/biophysj.106.094508 PMC1853137

[74] Subramanian VG, Duffy KR, Turner ML, Hodgkin PD. 2008. Determining the expected variability of immune responses using the cyton model. J. Math. Biol 56(6):861–92 17982747 10.1007/s00285-007-0142-2

[75] Hawkins ED, Markham JF, McGuinness LP, Hodgkin PD. 2009. A single-cell pedigree analysis of alternative stochastic lymphocyte fates. PNAS 106(32):13457–62 19633185 10.1073/pnas.0905629106 PMC2715326

[76] Markham JF, Wellard CJ, Hawkins ED, Duffy KR, Hodgkin PD. 2010. A minimum of two distinct heri-table factors are required to explain correlation structures in proliferating lymphocytes. J. R. Soc. Interface 7(48):1049–59 20053654 10.1098/rsif.2009.0488 PMC2880079

[77] Duffy KR, Wellard CJ, Markham JF, Zhou JH, Holmberg R, 2012. Activation-induced B cell fates are selected by intracellular stochastic competition. Science 335(6066):338–41 22223740 10.1126/science.1213230

[78] Hodgkin PD. 2018. Modifying clonal selection theory with a probabilistic cell. Immunol. Rev 285(1):249–62 30129201 10.1111/imr.12695 PMC6446824

[79] Asquith B, De Boer RJ. 2016. How lymphocytes add up. Nat. Immunol 18(1):12–13 27984568 10.1038/ni.3636

[80] Eickhoff S, Brewitz A, Gerner MY, Klauschen F, Komander K, 2015. Robust anti-viral immunity requires multiple distinct T cell-dendritic cell interactions. Cell 162(6):1322–37 26296422 10.1016/j.cell.2015.08.004 PMC4567961

[81] Brewitz A, Eickhoff S, Dähling S, Quast T, Bedoui S, 2017. CD8^+^ T cells orchestrate pDC-XCR1^+^ dendritic cell spatial and functional cooperativity to optimize priming. Immunity 46(2):205–19 28190711 10.1016/j.immuni.2017.01.003 PMC5362251

[82] De Boer RJ, Perelson AS. 2005. Estimating division and death rates from CFSE data. J. Comp. Appl. Math 184:140–64 10.1007/s11538-006-9094-8 16832737

[83] Yoon H, Legge KL, Sung SS, Braciale TJ. 2007. Sequential activation of CD8^+^ T cells in the draining lymph nodes in response to pulmonary virus infection. J. Immunol 179(1):391–99 17579060 10.4049/jimmunol.179.1.391

[84] Yoon H, Kim TS, Braciale TJ. 2010. The cell cycle time of CD8^+^ T cells responding in vivo is controlled by the type of antigenic stimulus. PLOS ONE 5(11):e15423 21079741 10.1371/journal.pone.0015423 PMC2975678

[85] Kretschmer L, Buchholz VR. 2022. T cell memories of past divisions. Nat. Immunol 23(5):646–47 35449418 10.1038/s41590-022-01193-3

[86] Henning AN, Roychoudhuri R, Restifo NP. 2018. Epigenetic control of CD8^+^ T cell differentiation. Nat. Rev. Immunol 18(5):340–56 29379213 10.1038/nri.2017.146 PMC6327307

[87] Weber TS, Perie L, Duffy KR. 2016. Inferring average generation via division-linked labeling. J. Math. Biol 73(2):491–523 26733310 10.1007/s00285-015-0963-3

[88] Schlub TE, Venturi V, Kedzierska K, Wellard C, Doherty PC, 2009. Division-linked differentiation can account for CD8^+^ T-cell phenotype in vivo. Eur. J. Immunol 39(1):67–77 19130548 10.1002/eji.200838554

[89] Karrer U, Sierro S, Wagner M, Oxenius A, Hengel H, 2003. Memory inflation: continuous accumulation of antiviral CD8^+^ T cells over time. J. Immunol 170(4):2022–29 12574372 10.4049/jimmunol.170.4.2022

[90] Sylwester AW, Mitchell BL, Edgar JB, Taormina C, Pelte C, 2005. Broadly targeted human cytomegalovirus-specific CD4^+^ and CD8^+^ T cells dominate the memory compartments of exposed subjects. J. Exp. Med 202(5):673–85 16147978 10.1084/jem.20050882 PMC2212883

[91] Kratchmarov R, Magun AM, Reiner SL. 2018. TCF1 expression marks self-renewing human CD8^+^T cells. Blood Adv. 2(14):1685–90 30021780 10.1182/bloodadvances.2018016279 PMC6058237

[92] Zhou X, Yu S, Zhao DM, Harty JT, Badovinac VP, Xue HH. 2010. Differentiation and persistence of memory CD8^+^ T cells depend on T cell factor 1. Immunity 33(2):229–40 20727791 10.1016/j.immuni.2010.08.002 PMC2928475

[93] Jeannet G, Boudousquié C, Gardiol N, Kang J, Huelsken J, Held W. 2010. Essential role of the Wnt pathway effector Tcf-1 for the establishment of functional CD8 T cell memory. PNAS 107(21):9777–82 20457902 10.1073/pnas.0914127107 PMC2906901

[94] Akondy RS, Johnson PL, Nakaya HI, Edupuganti S, Mulligan MJ, 2015. Initial viral load determines the magnitude of the human CD8 T cell response to yellow fever vaccination. PNAS 112(10):3050–55 25713354 10.1073/pnas.1500475112 PMC4364194

[95] Smith CJ, Venturi V, Quigley MF, Turula H, Gostick E, 2020. Stochastic expansions maintain the clonal stability of CD8^+^ T cell populations undergoing memory inflation driven by murine cytomegalovirus. J. Immunol 204(1):112–21 31818981 10.4049/jimmunol.1900455 PMC6920548

[96] Yates AJ. 2014. Theories and quantification of thymic selection. Front. Immunol 5:13 24550908 10.3389/fimmu.2014.00013 PMC3912788

[97] Crowl JT, Heeg M, Ferry A, Milner JJ, Omilusik KD, 2022. Tissue-resident memory CD8^+^ T cells possess unique transcriptional, epigenetic and functional adaptations to different tissue environments. Nat. Immunol 23(7):1121–31 35761084 10.1038/s41590-022-01229-8 PMC10041538

[98] Milner JJ, Toma C, Yu B, Zhang K, Omilusik K, 2017. Runx3 programs CD8^+^ T cell residency in non-lymphoid tissues and tumours. Nature 552(7684):253–57 29211713 10.1038/nature24993 PMC5747964

[99] Szabo PA, Miron M, Farber DL. 2019. Location, location, location: tissue resident memory T cells in mice and humans. Sci. Immunol 4(34):eaas9673 30952804 10.1126/sciimmunol.aas9673 PMC6778482

